# Canine Amniotic Fluid at Birth Holds Information about Neonatal Antibody Titres against Core Vaccine Viruses

**DOI:** 10.3390/vetsci11060234

**Published:** 2024-05-23

**Authors:** Debora Groppetti, Alessandro Pecile, Joel Filipe, Federica Riva, Alessia Inglesi, Pietro Andrea Kuhn, Elisa Giussani, Paola Dall’Ara

**Affiliations:** Dipartimento di Medicina Veterinaria e Scienze Animali, Università degli Studi di Milano, 26900 Lodi, Italy; debora.groppetti@unimi.it (D.G.); alessandro.pecile@unimi.it (A.P.); federica.riva@unimi.it (F.R.); alessia.inglesi@unimi.it (A.I.); pietroandrea.kuhn@studenti.unimi.it (P.A.K.); elisa.giussani@unimi.it (E.G.); paola.dallara@unimi.it (P.D.)

**Keywords:** dog, amniotic fluid, immunoglobulins, parvovirus, hepatitis, distemper

## Abstract

**Simple Summary:**

Due to its promising applications in diagnosis and therapy, amniotic fluid may represent the substrate of the future in obstetric and regenerative medicine. In this study, we explored its potential impact on canine neonatal immunity by investigating, in both maternal plasma and amniotic fluid collected at birth, total and specific immunoglobulins G against the three viruses responsible for most of the neonatal mortalities in dogs: canine parvovirus (CPV-2), infectious canine hepatitis virus (CadV-1), and canine distemper virus (CDV). Our findings revealed that both total and specific plasma maternal IgG titres were not strictly related to vaccination status, whereas specific immunoglobulin G concentrations in amniotic fluids showed some correlation with the bitch vaccination status. Furthermore, puppies that developed pathological conditions (i.e., diarrhoea of any origin) within the first two months of life exhibited significantly lower amniotic CAdV-1 antibody titres compared to healthy ones. The evaluation of antibodies in amniotic fluid at birth could provide crucial information on the actual immune status of newborns.

**Abstract:**

There is a growing interest in the composition of amniotic fluid (AF) in both humans and animals. In addition to its nutritional and protective functions for the foetus, current knowledge demonstrates that AF also serves advanced diagnostic, prognostic, and therapeutic roles. Newborn dogs have an underdeveloped immune system, making them highly susceptible to dangerous pathogens such as canine parvovirus (CPV-2), canine infectious hepatitis virus (CAdV-1), and canine distemper virus (CDV), thus exposing them to a high risk of mortality in the first weeks of life. Immunoglobulins G (IgGs) represent the only antibody isotype capable of crossing the placenta in a small amount and have been detected also in canine AF. The primary aim of this study was to investigate the reliability of AF collected at birth as a marker of passive immunity in canine species. For this purpose, total and specific IgGs against CPV-2, CAdV-1, and CDV were investigated and quantified in both maternal plasma and AF collected at the time of caesarean section. The vaccination status of the bitches was also taken into consideration. Since the immune system can be influenced by gestational age, with preterm infants having immature innate and adaptive immunity, IgG concentrations were correlated with amniotic lecithin, sphingomyelin, cortisol, surfactant protein A, and pentraxin 3 levels. In a previous study from our group on foetal maturity these molecules were measured in the same samples. Finally, correlations between their amniotic content and neonatal outcomes were investigated. This study demonstrates that AF analysis at birth can provide valuable insights into neonatal immunity in puppies, offering a non-invasive method to detect potential early health risks, for improved puppy care and management.

## 1. Introduction

The innate immune system begins developing during the intra-uterine period, whereas the adaptive immune system develops after birth and remains very immature in the puppy until 2–6 month of age. However, the passive immunity transmitted by the mother during pregnancy and especially through colostrum (the so-called Maternally Derived Antibodies, MDAs) ensures their survival [1,2]. 

The adaptive or acquired immune system involves specific responses to pathogens through lymphocytes that develop immunological memory against previously encountered antigens [1]. The innate immune system tends to be more mature at birth than the adaptive immune system, even though neither is fully developed until several weeks or months after birth [3]. 

During intra-uterine life, the placenta defends the embryo and foetus from exposure to foreign pathogens, and amniotic fluid (AF) modulates the maternal immune response to prevent the rejection of the foetus [4]. In dogs, the neonatal period is a critical age, with infectious diseases described as the primary cause of newborn mortality [5,6]. 

Vaccination stands as the primary tool for preventing dangerous and widespread diseases in both humans and domestic animals. Guidelines from different international associations categorize pet vaccines into core and non-core ones. Core vaccines are essential for all dogs, irrespective of lifestyle, as they provide protection against severe, hazardous, and potentially life-threatening diseases. Non-core vaccines are those recommended for some dogs based on lifestyle, geographic location, and risk of exposure. Due to the severity of their symptoms, parvovirus infection (CPV-2), infectious canine hepatitis (CAdV-1), and distemper (CDV) represent a widespread risk of canine neonatal morbidity and mortality, so the vaccines against these diseases are considered as core for all dogs worldwide [7,8,9]. 

In puppies, passive immune transfer occurs mainly through colostrum intake [10,11]. Indeed, the endotheliochorial placenta of carnivores forms an almost impenetrable barrier, allowing the passage of only about 5–12% of immunoglobulins (only IgGs) from the mother [10]. Despite their high molecular weight, IgGs are the only class of immunoglobulins capable of crossing the canine placenta and reaching the foetal circulation, even if in a small amount [12]. IgGs are transferred during the last twenty days of pregnancy in dogs [13], and they can be detected in the amniotic fluid at birth [10]. Gestational age at birth can influence the immune response, with preterm and early infants showing an increased risk of neonatal infections due to inefficient innate and adaptive immune systems [14,15,16]. A strong relationship between total IgGs and gestational age has also been reported in babies [16]. Due to the stressful impact of diagnostic procedures such as blood sampling, the latter is reserved in case of real emergency need, while non-invasive alternatives are strongly recommended in newborn puppies. 

In women, AF is a terrific matrix for early diagnosis of pregnancy-related abnormalities [17]. In the last few years, also in dogs, AF collected at birth has proven to be a potential and ethically appropriate diagnostic resource [18]. Indeed, AF contains lecithin, sphingomyelin, cortisol, and surfactant protein A (SP-A), which are considered predictive of foetal lung maturity in humans [19,20,21,22,23,24,25,26] and have recently been measured and correlated with gestational age in dogs [27]. Pentraxin-3 (PTX3) also was detected in AF; it is a soluble protein belonging to a superfamily of humoral mediators of innate immunity [28,29,30,31]. In women, PTX3 is regarded as an early marker of placental dysfunction [32] and is related to intrauterine foetal growth restriction [33], miscarriage [34], preeclampsia [35], and gestational age [36]. In veterinary medicine, this protein is still understudied, and a single study performed in canine species reported a positive correlation between amniotic PTX3 expression and gestational age [27]. 

Our hypothesis is that amniotic fluid holds information about the prenatal immune status of puppies that is reasonably related to maternal immunization while also providing early warnings for pathological conditions. 

To address this hypothesis, the first aim of the study was to measure total and specific IgGs against CPV-2, CAdV-1, and CDV in AF collected at the time of extraction of each puppy during elective caesarean section. Afterwards, the amniotic concentrations of total and specific IgGs for these pathogens were compared to their values in maternal plasma, also considering the vaccination status of the bitches. 

To further investigate the possible function of amniotic fluid in providing information on canine neonatal immunity, these total and specific IgGs measured both in AF and maternal plasma collected at birth were correlated to the content of amniotic lecithin, sphingomyelin, cortisol, SP-A, and PTX3. The concentrations of these molecules present in the AF (lecithin, sphingomyelin, cortisol, SP-A, and PTX3) was presented as part of a study on foetal maturity [27]; however, in the present study, they are correlated with specific immune parameters. 

Finally, all the molecules expressed in the amniotic fluid were compared with clinical features such as maternal age and body weight, litter size, neonatal sex, birthweight, viability (assessed by Apgar score), and morbidity and mortality of puppies within 2 months of life.

## 2. Materials and Methods

This study is part of a major project on canine amniotic fluid (Linea 2 Groppetti_2016) approved by the Ethical Committee of the Università degli Studi di Milano (OPBA_77_2017) and complied with Italian animal experimentation and ethics laws. Amniotic fluid was divided into two aliquots. The first aliquot was used for the analysis of amniotic lecithin, sphingomyelin, cortisol, SP-A concentration, and PTX3 gene expression levels in a previously published study on foetal maturity [27]; in this previous study, the concentrations of these molecules were published and were also correlated with foetal maturity parameters [27]. The second aliquot was used in the present project to evaluate amniotic lecithin, sphingomyelin, cortisol, SP-A concentrations, and PTX3 gene expression levels, as well as IgG titres. In the present study, only the correlations among these AF molecules and immunological and clinical parameters are shown.

### 2.1. Clinical Records

Ten purebred bitches scheduled for elective caesarean section were enrolled in this study. Surgical and anaesthetic protocols were performed as routine [37]. Only healthy bitches, based on clinical, ultrasonographic, and blood chemistry outcomes, were included.

Maternal clinical outcomes are summarized in Table 1. Four out of ten bitches had regular prophylaxis with core vaccines (ID. 1,4,8,9).

All dogs were monitored throughout the reproductive cycle, from proestrus to parturition, as routine [38]. Anamnestic and clinical data, such as age, body weight, breed, and vaccination status, were recorded. Bitches were either mated or inseminated and fed with commercial diets specific for lactating dogs (from mid-pregnancy to weaning) of different brands. Surgery was planned when foetuses were considered mature based on accurate gestational age estimation, as reported in the literature [38,39,40]. Litter size, sex, birth weight, viability, and morbidity (regardless of the cause) and mortality of puppies within the first 2 months of life were recorded. Immediately after extraction, each puppy received first neonatal care and/or emergency rescue if needed, following the recommended procedures [41]. Briefly, within the first 5 min of life, neonatal viability was assessed in each puppy using a specific Apgar score ranging from 0 to 14 to classify them as healthy, moderately stressed, or severely stressed [42]. All puppies were fed colostrum and maternal milk, with some artificial milk supplementation as needed in large litters.

### 2.2. Maternal Blood Collection

Just before undergoing caesarean section, 1 mL of venous blood was collected from the cephalic vein of the bitches under general anaesthesia. Whole blood was placed into K2EDTA tubes, and the plasma samples obtained after centrifugation at 1500× *g* for 10 min at room temperature were stored at −20 °C until immunoglobulins analysis.

### 2.3. Amniotic Fluid Collection

AF was collected at the time of extraction of each puppy during elective caesarean section as previously described [27]. The fluid was aspirated by a 20 mL sterile syringe that delicately pierced the wall of the amniotic sac. The puppy was held upright with the head up and the needle inserted in the most declivous portion to avoid injuring the puppy with the syringe needle. Each amniotic sample was aliquoted into two tubes of 15 mL and immediately centrifuged at 500× *g* for 15 min at room temperature, and the supernatant was stored at −80 °C until analysis.

### 2.4. Amniotic Fluid Analysis

#### 2.4.1. Total IgGs

Concentrations of total Immunoglobulins G in the amniotic fluid were assessed using an ELISA method (Dog IgG Quantitation Set; Bethyl Laboratories, Inc., Montgomery, TX, USA). Ninety-six-well microtiter plates were coated with 100 µL/well of capture affinity-purified antibody (sheep anti-dog IgG) diluted 1:100 in coating buffer (carbonate-bicarbonate buffer, 0.05 M; pH 9.6) for 1 h at room temperature. After incubation, the plates were washed four times with Tris-buffered saline (TBS) containing 0.05% Tween 20 (TBST). Subsequently, the plates were coated with 200 µL/well of TBST for 30 min and washed again four times. Seven dilutions of reference dog antibodies ranging from 500 to 7.8 ng/mL were used. Based on established assays to determine optimal dilutions for expected results, amniotic fluids were diluted 1:5000. All dilutions were prepared in TBST. In each well, 100 µL of each reference serum or sample dilution were added in duplicate, incubated for 1 h at room temperature, and then washed four times before adding 100 µL/well of conjugate (sheep anti-dog IgG horseradish peroxidase [HRP] conjugate, diluted 1:100,000). The latter was incubated for 1 h at room temperature. The wells were washed five times before adding 100 µL/well of substrate chromogen (H_2_O_2_ and TMB) and left for 15 min in the dark; finally, the reaction was stopped by adding 100 µL/well of stop solution (sulfuric acid, 0.18 M), and the plates were read at 450 nm using an ELISA microplate reader (Thermo Fisher Scientific, Tokyo, Japan). The intra-assay and inter-assay coefficients of variation were 3.08% and 4.38%, respectively.

#### 2.4.2. Specific IgG—VacciCheck

Each plasma and AF sample underwent analysis using the in-clinic test Canine VacciCheck (Biogal, Kibbutz Galed, Israel, distributed in Italy by Agrolabo, Scarmagno, Italy), in accordance with the manufacturer’s instructions. The kit employs a dot-ELISA-based rapid semi-quantitative system that is approved for measuring specific antibody titres (IgGs) against CPV-2, CadV-1, and CDV. VacciCheck demonstrates high specificity and sensitivity for each virus and holds approval for both research and diagnostic applications. In this test, the antibody concentration is determined by the colour intensity of the resulting spots, compared with a scale ranging from 1 to 6. The S0 value, standardised by the manufacturer, is considered equivalent to an antibody titre of <1:20 for CPV-2, <1:4 for CAdV-1, and <1:8 for CDV. Meanwhile, an S value of 3 (S3) is defined as equivalent to 1:80 for CPV-2, 1:16 for CAdV-1, and 1:32 for CDV. A value equal to or higher than S3 indicates that the individual is protected against each of these three diseases.

#### 2.4.3. Lecithin, Sphingomyelin, Cortisol, SP-A, and PTX3 Detection in Amniotic Fluid

As previously published by our team, lecithin and sphingomyelin were measured using an HPLC-MS method (Thermo Q-Exactive Plus, Thermo Scientific), cortisol with a quantitative test based on the ELFA technique (Enzyme Linked Fluorescent Assay, MiniVidas, bioMérieux, Bagno a Ripoli (FI), Italy), SP-A with a commercial sandwich ELISA assay kit (LifeSpan BioSciences, Seattle, WA, USA), and PTX3 mRNA in the AF cell pellet was measured by qPCR [27].

### 2.5. Statistical Analysis

Maternal age and body weight were analysed as both continuous and categorical variables, as described below: age (≤3 years; >3 years), body weight (≤30 kg; >30 kg). Bitches that regularly received the core vaccine according to the WSAVA guidelines [7] were defined as “regularly vaccinated”, while those with an irregular protocol were deemed “irregularly vaccinated”. Neonatal mortality was recorded at birth, within 7 days, and at 2 months of life. Puppies affected by pathological conditions of any cause during the 2-month observation period were classified as “pathological”, while puppies without any clinical signs were deemed as “healthy”. Since the only clinical symptom observed in our caseload was diarrhoea, “pathological” puppies refer to those with diarrhoea. Statistical analyses were performed using GraphPad Prism 6 (La Jolla, CA, USA), considering statistically significant values at *p* < 0.05. Descriptive statistics were expressed as mean ± standard error. The Shapiro–Wilk test was used to verify the distribution of data. Based on normal or not-normal distribution of the data, parametric (two-tail Pearson correlation) or non-parametric (two-tail Spearman correlation) tests were used to check correlations (bivariate linear correlations) among maternal and puppies’ parameters. To compare two experimental groups based on normal or not-normal distribution of the data, parametric (Student *t* test) or non-parametric (Mann–Whitney test) tests were used, respectively.

#### Experimental Design

This study assessed three different conditions retrospectively: the type of vaccination administered to the bitches, the antibody (Ig) titres of the mothers for each core vaccine, and the health condition of the puppies during the two-month follow-up period.

Regarding vaccination type, the mothers were categorized into two groups: regularly vaccinated, where mothers received all core vaccines at regular intervals as recommended by vaccine manufacturers, either annually or every two to three years; and irregularly vaccinated, where mothers did not receive core vaccines at the prescribed regular intervals.

For Ig titres related to each core vaccine, the mothers were classified into two groups: low titres, indicating an antibody titre lower than scale value 3 (S3), and high titres, with an antibody titre equal to or higher than S3 (based on vaccicheck scale). IgG titres for each vaccine were measured using the Vaccicheck kit (Biogal, Kibbutz Galed, Israel, and supplied in Italy by Agrolabo, Scarmagno, Italy), comparing unknown samples with a scale ranging from 0 to 6. In the kit, the S3 value is regarded as the protective titre, corresponding to an antibody titre of 1:80 for CPV-2, 1:16 for CAdV-1, and 1:32 for CDV.

Concerning the puppy health conditions, newborns were divided into two groups: healthy (puppies that did not present any clinical sign—diarrhoea—during the follow-up period) and pathological (puppies that presented clinical signs—diarrhoea—during the follow-up period).

## 3. Results

### 3.1. Clinical Outcomes

A total of 63 puppies were born, comprising 34 males and 29 females, all of which were born alive. The birthweights varied from 236 gr to 770 gr (438.5 ± 140.5). Apgar scores ranged between 4 and 14 (10.9 ± 2.3). Unfortunately, eight puppies died within the first 48 h of life. Among them, one anasarca puppy survived only a few hours due to serious health conditions, two puppies were accidentally crushed to death by the dams, and five puppies died from causes that remained uninvestigated but were possibly attributed to incorrect management by the owners. The remaining 55 puppies survived until the last follow-up at 2 months. 

Eighteen out of these 55 puppies from five bitches showed diarrhoea of varying intensity during the observational period and were included in the pathological group. Since a diagnosis of the specific etiological cause (i.e., infectious, nutritional) was not reached, we considered puppies affected by diarrhoea of any origin. In particular, the entire litter of three bitches (the two French Bouledogue dogs belonging to the same owner and living together, and the Bernese Mountain dog), i.e., a total of 16 puppies, presented diarrhoea. The remaining 37 puppies were considered healthy, since no clinical signs were observed.

### 3.2. Immunization State

Total and specific immunoglobulins were detected and titrated in all maternal plasma and amniotic samples, except for two puppies from the same litter (ID.5), for which the amount of AF collected at birth was insufficient. Total plasma and amniotic IgG concentrations varied from 5.6 to 14.1 mg/mL (10.2 ± 2.8) and 0.02 to 0.5 mg/mL (0.1 ± 0.09), respectively, and were not significantly correlated with each other.

**Figure 1:**
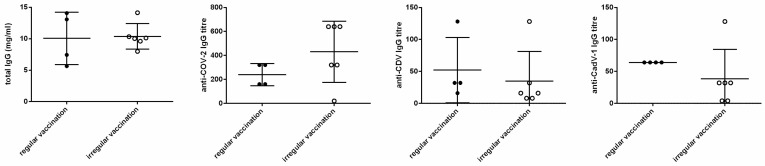
Influence of regular (n = 4) or irregular (n = 6) vaccination on IgG concentration and titres in maternal blood.

**Figure 2:**
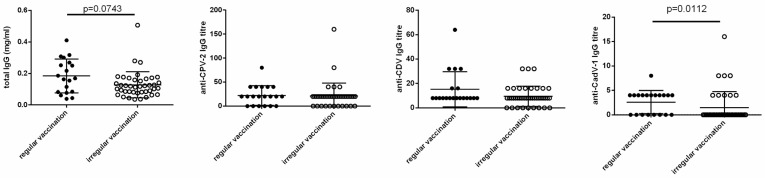
Influence of regular (n = 20) or irregular (n = 43) vaccination on IgG concentration and titres in the amniotic fluid.

Total IgG plasma concentration was similar in regularly vaccinated (10.0 ± 4.1 mg/mL) and irregularly vaccinated bitches (10.4 ± 2.0 mg/mL) without statistically significant differences (Figure 1). In amniotic fluid at birth, total IgG tended (*p* = 0.07) to be higher in litters of regularly vaccinated mothers (0.18 ± 0.1 mg/mL) than in litters of irregularly vaccinated ones (0.13 ± 0.08 mg/mL, Figure 2).

Titres of specific immunoglobulins G against CPV-2, CAdV-1, and CDV in maternal plasma and amniotic fluid are shown in Table 2.

Specific IgG titres against CPV-2, CAdV-1, and CDV were similar in the plasma of regularly and irregularly vaccinated bitches, as shown in Figure 1. In amniotic fluid, IgGs against CAdV-1 (*p* = 0.01), but not against CPV-2 or CDV, were higher in regularly vaccinated than irregularly vaccinated litters (Figure 2 and Figure S1). 

Table 3 shows the threshold levels (S3) of antibody titres considered protective against each of these three viruses in canine plasma [43] and the number of dams with protective antibody titres. Bitches were classified based on their antibody titre against each disease. In particular, bitches with an antibody titre lower than the threshold level (S3), i.e., the minimum protective IgG value, were considered “low-titre bitches”; whereas bitches with an antibody titre equal to or higher than the threshold level (S3) as “high-titre bitches”.

**Figure 3:**
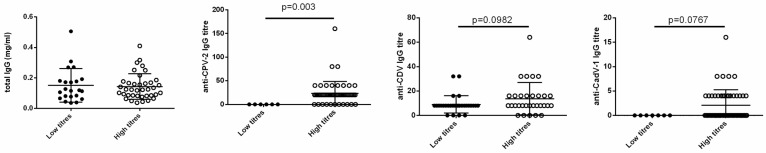
Comparison of amniotic antibody titres against CPV-2, CAdV-1, and CDV between dams with high and low antibody titres.

Four out of ten bitches (three regularly vaccinated and one irregularly vaccinated) had serological protective titres against all the three viruses. When considering high- and low-titre-value dams (i.e., bitches with or without protective IgG values—S3), concentrations of IgG against CPV-2 were higher in the amniotic fluid of puppies born from mothers with high antibody titres than in those born from dams with lower ones (*p* = 0.003, Figure 3). Though without significance, amniotic antibodies against CAdV-1 (*p* = 0.08) and CDV (*p* = 0.09) tended to be higher in puppies born from high-antibody-titre mothers than low-titre ones.

Among the amniotic components (i.e., lecithin, sphingomyelin, cortisol, SP-A, and PTX3), only sphingomyelin negatively correlated with total IgG amniotic concentration (*p*-value = 0.0057). Amniotic cortisol positively correlated with anti-CDV IgG titres in amniotic fluid (*p*-value = 0.0358). Finally, PTX3 expression in amniotic fluid negatively correlated with anti-CadV1 IgG titres in amniotic fluid (*p*-value = 0.0136).

### 3.3. Statistical Correlations with Clinical Features

**Figure 4:**
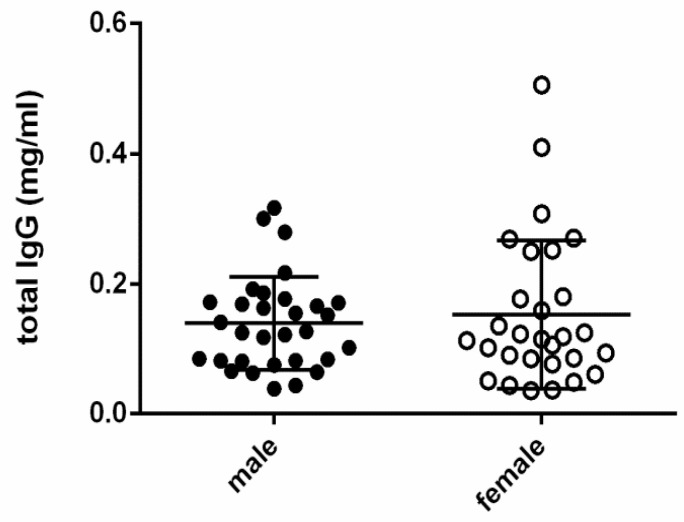
Comparison of total IgG titres in AF between male (n = 34) and female (n = 29) puppies.

Maternal age, body weight, and litter size showed some correlations with antibody concentrations either in plasma or amniotic fluid and even with amniotic components. Actually, maternal age positively correlated with amniotic SP-A values (*p*-value = 0.0101), body weight and litter size both negatively correlated with amniotic cortisol levels (*p*-value = 0.0004 and 0.0055, respectively), and maternal age, body weight, and litter size were also correlated with specific IgG against CPV-2 (*p*-value < 0.0001 for all parameters)—maternal age negatively, and the other two parameters positively. We also compared male and female puppies, but there were no differences in total and/or specific amniotic IgG levels between newborn puppies of different genders (Figure 4).

**Figure 5:**
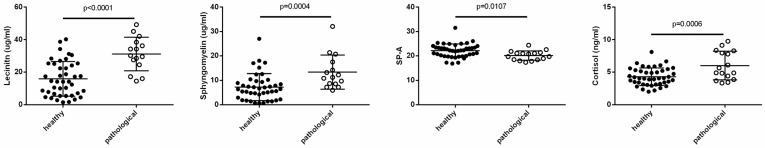
Different expression of AF molecules between healthy (n = 37) and pathological (n = 18) puppies.

Also, birthweight, Apgar score, and neonatal mortality did not significantly influence the IgG concentrations. Interestingly, puppies in the pathological group had higher values of amniotic lecithin (*p* = 0.0001), sphingomyelin (*p* = 0.0004), and cortisol (*p* = 0.0006) and lower values of SP-A (*p* = 0.0107) than healthy puppies (Figure 5).

**Figure 6:**
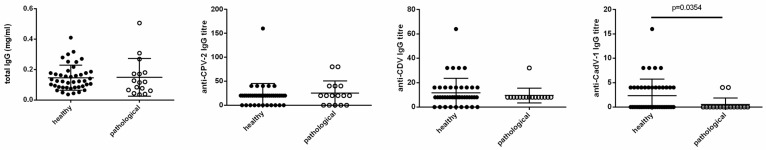
Specific and total IgG titres in healthy (n= 37) and pathological (n = 18) puppies.

Pathological puppies showed also lower IgG titres against CAdV-1 (*p* = 0.035) than healthy puppies (Figure 6).

**Figure 7:**
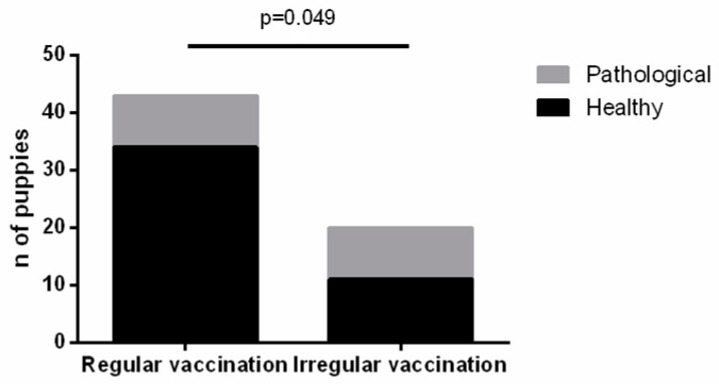
Vaccination of the dams and health status of the puppies.

It is interesting to note that the health status of puppies is somehow related to the vaccination status of the dam. We observed that the number of puppies with pathology is statistically higher among litters from dams that are not regularly vaccinated (*p*-value = 0.049) (Figure 7).

## 4. Discussion

Amniotic fluid (AF) performs crucial and diverse functions, including protection of the foetus from trauma and temperature changes, as well as contribution to the development and maturation of the foetal respiratory and digestive systems [44]. In humans, AF plays a diagnostic role in early detection of infections, genetic and pregnancy disorders, and birth defects [45]. More recently, it has been demonstrated as a promising therapeutic application in regenerative medicine [46].

Collection of the amniotic fluid at birth in dogs through caesarean section is a simple, safe, non-invasive, accurate, and rapid method that does not compromise the puppy’s extraction time. In our study, AF sampling was successful in all 63 puppies, although its amount was insufficient in two littermates, possibly due to a high litter number (11 puppies) for a medium-sized breed. It is plausible to assume an inverse relationship between AF volume and litter size, an aspect warranting further investigation as, to the best of our knowledge, it has not yet been reported. 

Our results demonstrated the presence of total and specific immunoglobulins G against CPV-2, CAdV-1, and CDV in canine AF at birth. Since the foetus swallows amniotic fluid containing immunoglobulins, and a possible systemic transfer via intestine absorption may occur, we speculate that IgG concentration in amniotic fluid is positively correlated with that in the blood of newborn puppies, as reported in humans [47]. 

Understanding the real immune status of newborns allows for informed assessment of their infectious risks and facilitates planning the puppy’s vaccination program. In fact, the immune response to vaccination, even when correctly performed, varies and is influenced by multiple factors [11,48,49,50,51]. Maternally Derived Antibodies (MDAs) are transferred from mothers to puppies during pregnancy and continue after delivery with colostrum assumption. Consequently, for the first weeks of life, puppies should be protected by MDAs, which can show titres with a significant individual variability [11,50,51]. MDAs are considered a double-edged sword, since they are essential for the puppies’ survival but heavily interfere with vaccination, given that as long as MDA titres are high, eventually vaccination fails [6,11,51,52]. 

Pregnancy is a unique state where maternal adaptive immune response is reduced to tolerate the foetus and placenta [53], while the activity of innate immunity is increased allowing maintenance of immune function homeostasis [54]. Our study revealed no correlation between total and specific IgG titres in the plasma of mothers and their vaccination status. Therefore, it is advisable to test core vaccine titres in bitches prior to breeding, even if they receive routine vaccinations [55]. In our caseload, the percentage of dogs with protective antibody titres against CPV-2 (90% vs. 86%) and CAdV-1 (80% vs. 71%) was similar to that of a previous study [56], while against CDV (50% vs. 72%), it was lower. This finding could depend on many different population-related factors, such as the number of dogs, sex, age, weight, breed, attitude, stress, or health status [2,11,56,57]. 

Transplacental immunoglobulin transfer is essential for newborns, whose immune system is still immature [53]. In dogs, the importance of passive immunity, in protecting against infections, neonatal morbidity, and mortality, has recently been highlighted [51]. Although data for dogs are lacking, the possibility of maternal antibodies directly crossing the placenta, as observed in humans, is considered [58]. However, our study did not find a correlation between maternal and amniotic levels of total IgGs, suggesting a potential share of foetal-origin IgGs or varying transplacental transfer efficiency among littermates. Total amniotic IgGs tended to be higher in litters of regularly vaccinated dams, even though not significantly (*p*-value = 0.07). 

Regarding the specific antibodies, IgGs against CAdV-1 were higher in the AF of regularly vaccinated dams (*p* = 0.01), and IgGs against CPV-2 were higher in the AF of dams with higher antibody titres (*p* = 0.003). IgGs against CAdV-1 and CDV showed the same, though not significant, tendency to be higher in the AF of high-titre than low-titre bitches. These results suggest different percentages of specific IgG transport through the placenta from the maternal circle to the foetal one or a different half-life of the IgG against different viruses after the vaccination [7,11,48,59]. 

Rather than maternal plasma, AF appeared to hold information about neonatal immunity with interesting diagnostic potential, allowing for early screening of the immune condition of newborn puppies and their effective protection from infectious diseases. Furthermore, puppies that developed gastrointestinal symptoms during the 2-month follow-up period had lower titres of IgG against CAdV-1 than healthy puppies (*p* = 0.04). 

Our previous research demonstrated the presence of lecithin, sphingomyelin, cortisol, SP-A, and PTX3 in canine amniotic fluid [27]. Some of these amniotic components showed some correlation with total and specific IgGs and also with some neonatal parameters. 

The positive correlation that emerged from our results between amniotic cortisol and specific IgG against CDV is difficult to frame in a clinical setting and certainly deserves further investigation, as there is no reference literature on this specific aspect. In general, it could fit with the concept that temporary stressors of a mild to moderate nature, i.e., leading to a high amniotic cortisol, enhance immunity, thus increasing IgG levels [60]. On the contrary, chronic stress is reported to compromise neonatal immunity [61].

We also described a negative correlation between amniotic sphingomyelin and the concentration of total IgG in AF that could be associated with the regulatory activity of sphingomyelin on the CD-1d antigen presentation to T and NK cells [62]; the negative correlation between amniotic PTX3 expression and anti-CadV1 IgG titres should be further investigated.

So far, the impact of maternal age on amniotic SP-A, lecithin, and sphingomyelin concentrations has been rarely investigated, and very little is known even in human medicine. In women, a negative effect of ageing on surfactant protein-A and a variable trend between age and lecithin concentration in blood have been reported, which both are the opposite of what we found in dogs [63,64]. However, more research is needed to better understand their clinical implications in dogs.

A significant increase of lecithin (*p* = 0.0003), sphingomyelin (*p* = 0.0008), and cortisol (*p* = 0.01) concentrations was noticed in the AF of pathological puppies compared to healthy ones. A positive correlation between amniotic lecithin, sphingomyelin, and cortisol concentrations has already been reported both in humans [65] and in dogs [27]. We can speculate that a high amniotic cortisol level, as a marker of foetal distress, may predispose newborns to the development of pathological conditions, i.e., diarrhoea in our caseload, in accordance with the theory of foetal programming reporting that events occurring during uterine life are linked to future adult diseases [66]. It is, in fact, known that maternal stress can be responsible for an increased susceptibility to disease in babies [67]. Moreover, deficiencies in surfactant components may be responsible for lung and kidney alterations, with SP-A used as a disease biomarker in humans [68]. In accordance with the literature, the group of pathological puppies in our caseload showed lower SP-A values than healthy ones (*p* = 0.005). However, since the cause of the pathological manifestations was not diagnosed and many aspects, including maternal and neonatal nutrition, can determine the appearance of diarrhoea in newborns, these aspects deserve deeper investigation. 

The impact of dog breed and gestational age on variation in amniotic fluid composition also requires further study. In fact, women of different ethnicities show different amniotic immunomodulatory properties [69,70]. A similar state can also occur in dogs, thus affecting neonatal immunity, since some breeds are known to be genetically prone to infectious diseases [71]. Furthermore, a recent study underlined amniotic composition variation during pregnancy in dogs and cats [72].

Genetic and breed influence on cortisol levels has been reported in farm animals [73]. The negative correlation we observed between amniotic cortisol and both maternal body weight and litter size may depend on this influence, as large dog breeds are heavier and have larger litters than smaller dogs.

Another interesting result of this study was the positive correlation found between the maternal body weight and specific IgG against CPV-2 levels in the amniotic fluid. Dog size is a known factor influencing the strength of the immune response, with larger dogs generally receiving less protection than smaller breeds. Larger dogs may have a higher amount of subcutaneous fat at traditional vaccine injection sites, which could sequester vaccine antigens and limit their visibility to immune cells [74,75]. However, in two recent studies by Dall’Ara et al., it was demonstrated that larger dogs showed higher antibody titres against CPV-2 compared to medium and small breeds. The increased immune response against this specific virus might be attributed to environmental exposure, as CPV-2 can persist for extended periods in the environment. Consequently, larger dogs, which are often more active and kept outdoors or allowed to roam freely with their owners, have a higher likelihood of exposure to CPV-2 [76].

These findings emphasize the potential for amniotic fluid as a diagnostic tool for evaluating neonatal immunity and health in dogs, highlighting the need for further research to explore its practical applications and implications for breeding and veterinary care.

## 5. Conclusions

The collection of amniotic fluid (AF) at birth in canine puppies offers a reliable and non-invasive method to assess neonatal immunity and provides insights into the transplacental transfer of maternal antibodies (MDAs). In the near future, this approach may serve as an early warning system for potential future health issues in puppies. Further research into variations in AF composition across dog breeds and gestational ages could lead to a better understanding of neonatal immunity and its impact on puppy health.

## Figures and Tables

**Table 1:** Breed, age, body weight, and litter size of bitches enrolled in this study.

ID	Breed	Age (ys)	BW * (kg)	Litter Size
1	German Shepherd	7	34.5	8
2	German Shepherd	5	28.3	6
3	American Bully	2	23.8	2
4	American Bully	3	23.3	4
5	American Bully	2.5	38.5	11
6	Rhodesian Ridgeback	7	40.5	11
7	American Bully	2.5	28.5	5
8	French Bouledogue	3	12.5	6
9	French Bouledogue	3.5	11.4	2
10	Bernese Mountain Dog	3	56.2	8
	Mean ± SD	3.9 ± 1.8	29.7 ± 13.4	6.3 ± 3.2

* BW: body weight.

**Table 2:** Antibody titres against CPV-2, CAdV-1, and CDV in maternal plasma and amniotic fluid.

		Plasma				AF		
**CPV-2** ^1^		number of dams (n = 10)				number of puppies (n = 61)		
	min–max	≤1:20	1:160–320	>1:320	min–max	Negative	≤1:20	>1:20
	1:20–1:640	1	4	5	negative—1:40	17	33	11
**CAdV-1** ^2^		number of dams (n = 10)				number of puppies (n = 61)		
	min–max	≤1:4	≤1:32	>1:32		Negative	≤1:4	>1:8
	1:4–1:128	2	2	6	negative—1:4	39	16	6
		number of dams (n = 10)				number of puppies (n = 61)		
	min–max	≤1:8	≤1:16	>1:16		Negative	≤1:16	>1:16
**CDV** ^3^	1:8–1:128	2	3	5	negative—1:16	9	45	7

^1^ CPV-2: canine parvovirus; ^2^ CadV-1: infectious canine hepatitis virus; ^3^ CDV: canine distemper virus.

**Table 3:** Percentage of bitches with protective antibody titres against CPV-2, CAdV-1, and CDV.

Protective Threshold (S3)	CPV-2 ≥1:80 *	CAdV-1 ≥1:16 *	CDV ≥1:32 *
Percentage of regularly vaccinated (4) protected bitches	100	100	75
Percentage of irregularly vaccinated (6) protected bitches	83.3	66.7	33.3
Percentage of protected bitches out of the total (10)	90	80	50

* In bold are the protective IgG values.

## Data Availability

Data contained within the manuscript.

## Supplementary Materials

The following supporting information can be downloaded at: https://www.mdpi.com/article/10.3390/vetsci11060234/s1. Figure S1—Comparison between the immune status of the mother (Vaccination regular/irregular and low/high titers) and relationship with pathological puppies.
