# Supplementary material for: Canine Amniotic Fluid at Birth Holds Information about Neonatal Antibody Titres against Core Vaccine Viruses
Source: Vet Sci. 2024 May 23;11(6):234. doi: 10.3390/vetsci11060234 (PMC11209429; doi:10.3390/vetsci11060234)
Supplement: Supplementary file 1 [file vetsci-11-00234-s001.zip › vetsci-2968998-supplementary.pdf]

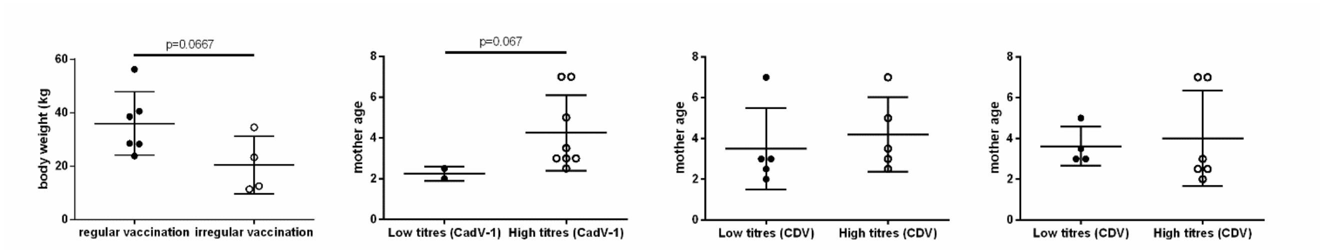

Figure S1 - Comparison between the immune status of the mother (Vaccination regular/irregular and low/high titers) and relationship with pathological puppies
